# Bioecology of Dominant Malaria Vector, *Anopheles superpictus* s.l. (Diptera: Culicidae) in Iran

**Published:** 2018-09-30

**Authors:** Hassan Vatandoost, Ahmad Ali Hanafi-Bojd, Ahmad Raeisi, Mohammad Reza Abai, Fatemeh Nikpour

**Affiliations:** 1 Department of Medical Entomology and Vector Control, School of Public Heath, Tehran University of Medical Sciences, Tehran, Iran; 2 Department of Chemical Pollutants and Pesticides, Institute for Environmental Research, Tehran University of Medical Sciences, Tehran, Iran; 3 Ministry of Health and Medical Education, Tehran, Iran

**Keywords:** *Anopheles superpictus* s.l., Ecology, Biology, Insecticide resistance, Iran

## Abstract

**Background:**

Malaria continues to be a main vector-borne public health problem in Iran. The endemic foci of the disease are mainly located in south-eastern part of the country. Iran is now launching the elimination of malaria. Studies on the bioecology and susceptibility of malaria vectors to insecticide are essential in this phase.

**Methods:**

The literature on bio-ecology of *Anopheles superpictus* s.l. was reviewed in Iran in more than half a century. Different aspects including, distribution, key identification, larval habitats, flight range, seasonal activities, irritability/susceptibility to insecticides, and anthropophilicity index were identified.

**Results:**

The adult females of *An. superpictus* s.l. were susceptible to all WHO-recommended imagicides except DDT. Distribution, morphology, sibling species, larval habitat, flight range, irritability tests, sustainability index, blood feeding preference and related factors were discussed in details

**Conclusion:**

Results of the evaluating will help for decision making of authorities for vector control.

## Introduction

Mosquito-borne diseases are the major problems worldwide, among them malaria presents a major health problem globally. In 2016, an estimated 216 million cases of malaria occurred worldwide, compared with 237 million cases in 2010 and 211 million cases in 2015. Most malaria cases in 2016 were reported from the WHO African Region, while the two percent of cases were inhabitants of WHO Eastern Mediterranean Region countries (1). It is one of the important infectious diseases in Iran with an average of about 15000 annual cases in the last decade, while total recorded cases has dropped to less than 100 locally transmitted cases in 2017. Most of indigenous and imported malaria cases in Iran are reported from southern and southeastern areas of the country in Sistan and Baluchistan, Hormozgan and South of Kerman Provinces. The most routes of malaria cases are immigration from Afghanistan and Pakistan to southern and southeastern areas of the country (Ministry of Health, annual reports).

Anopheline mosquitoes (Diptera: Culicidae) are vectors of malaria to humans. Currently there are proven and effective tools to fight against malaria including vector control measures (1). As these tools are scaled up, malaria endemic countries need to continually update the skills and competence of the health workers engaged in malaria control and elimination.

All observations indicate that the data reflect the real situation and that the overwhelming majority of cases, which occur, are included in the national system, although there is room for improvement in the surveillance system. The spectacular progress can be ascribed to effective implementation of appropriate curative and preventive control interventions through a strong health care infrastructure. Social and economic development allowing better housing, use of air-conditioning etc. has also played a role.

There are several activities on different aspects of malaria in the country: including insecticide resistance monitoring (2–12), sibling species, molecular study, new record (13–20), novel methods for vector control (21–26) faunestic study (27, 28), use of plants for larval control (29–41) using bednets and long lasting impregnated nets (42–48), morphological studies (49–51), malaria epidemiology (52–55), ecology of malaria vectors (56–64), biodiversity (65, 66), community participation (45, 54), vector control (67), repellent evaluation (68), anthropophilic index of malaria vectors (65, 69), training (70) is designated as malaria training center by WHO. There are several reports on different aspects of malaria vectors recently different studies have been conducted during more than 90 years on malaria (71–96) and its vectors in Iran.

Seasonal activity of Anopheline mosquitoes and their peak of activity vary in different area due to environmental condition. This issue affected the epidemiology of malaria transmission in different regions. Agriculture in Iran remains highly sensitive to climate developments, the country’s most important crops are wheat, rice and other grains, sugar beet, fruits, nuts, cotton, and tobacco, which require the use of insecticides. So far different groups of insecticides are using for crops protection in the country. The main governmental use of insecticide in the health sector is their application for adult mosquito control. The first attempts to control malaria vectors started during the 1960’s with organochlorines (DDT, dieldrin and BHC), followed by organophosphates (Malathion and pirimiphos-methyl) for about two decades from 1966. After that during 1977–1990 propoxur was used by national malaria vector control program. Subsequently, after the introduction of pyroteroids into the market, and given their lower risk for humans and more affordable products, lambdacyhalothrin and deltamethrin were used. As the adult mosquitoes control, larval control is also used to reduce the vector abundance. For this purpose, Temephos, Reldan® and pirimiphos-methyl was used in past decades.

## Materials and Methods

All the published papers thought the internet and master of sciences and PhD thesis related to *An. superpictus* s.l. were reviewed and evaluated. Key words for search in scientific motor engines were: *Anopheles superpictus*, insecticide resistance, ecology, distribution, identification, anthropophilicity, sporozoite rate, Iran.

## Results

### Distribution of *Anopheles superpictus* s.l. in Iran

**Fig. 1.**
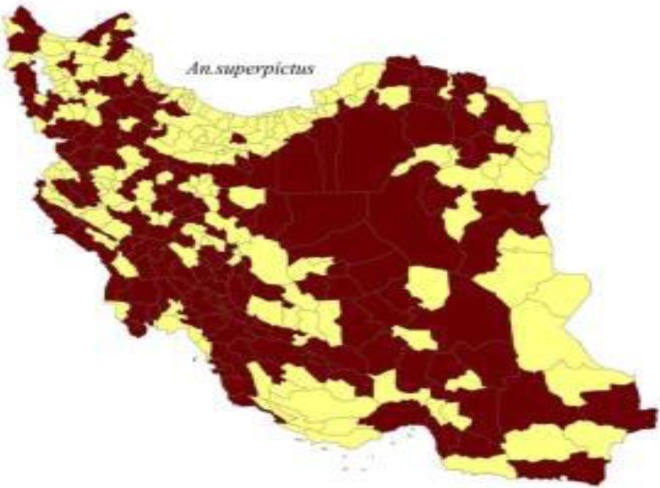
Distribution of *Anopheles superpictus* s.l. in Iran

Faghih’ research results showed that this species could be found in areas of 2000 meters above sea level (97–99). It has been captured from coastal plains of Persian Gulf in areas with 500 meters height above sea level (in Chelou village in Minab). With the exception of a narrow strip of coastal areas, this species is found in almost all regions of Hormozgan Province (100–104) (Fig. 1).

### Key to the female *Anopheles superpictus* s.l.

Wings with contrasting pale and dark spots, at least on costa (C), radius (R) and radius-one (R1), Hind tarsomeres 3–5 no1 entirely pale, Maxillary palpus dark, or with at most 3 distinct pale bands (pale spots may also be present): abdominal terga II–VII without dark scale-tulips. Although some posterolateral dark scales may be present on distal segments, Hind tarsomere 5 dark, Palpomere 5 entirely pale, Femora and tibiae not spotted: abdominal terga without pale scales, Anal vein with 2 dark spots, distal spot long (104).

### Key to the *Anopheles superpictus* s.l. larvae

Seta 2- Cephalon inserted at least as far apart as the distance between 2- Cephalon and 3- Cephalon on one side, seta 1- Antenna always simple, seta 5, 6, 7- Cephalon branched (subgenus Cellia), Tergal plates on abdominal segments III–VII smaller, < 0.5 width of segment, and not enclosing median accessory tergal plate, Thoracic pleural seta various, Thorax with at least long pleural seta 9- Prothorax and 9- Metathorax branched, 9, 10- Mesothorax, and 10- Metathorax simple or branched, Thorax with long pleural seta 9- Prothorax and 9, 10- Metathorax branched.

**Fig. 2.**
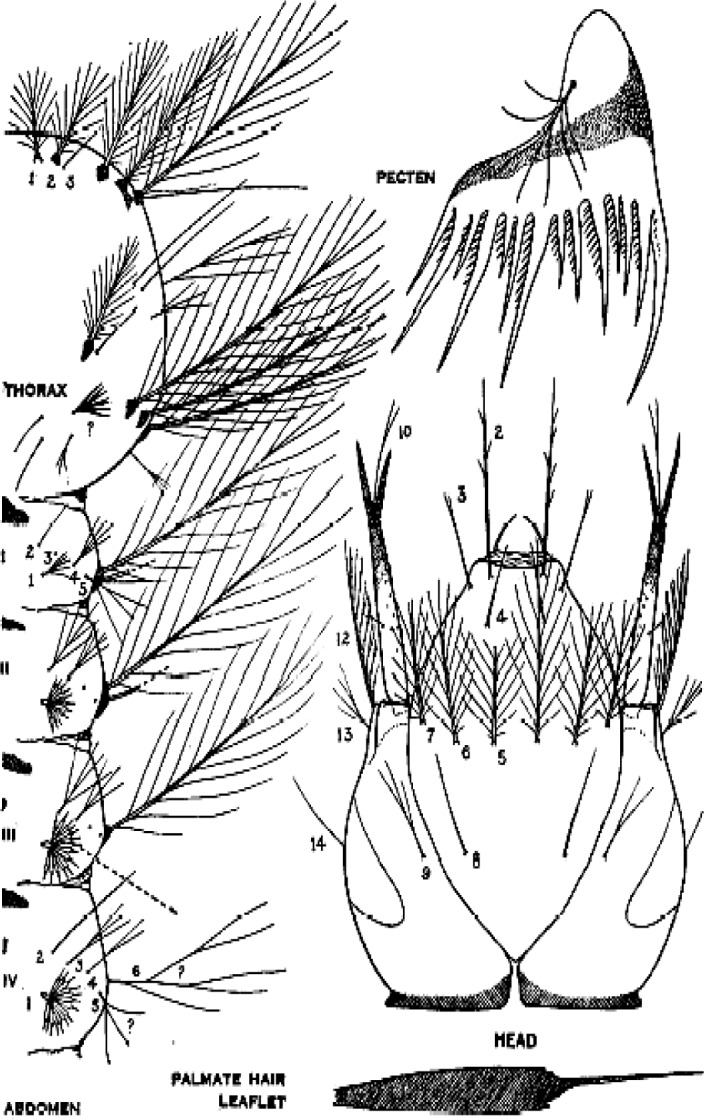
Morphology of larva of *Anopheles superpictus* s.l.

Seta 9- Mesothorax branched, 10- Mesothorax simple, palmate seta 1-III-VII strong, leaflets and filaments various, Seta 3- Cephalon simple or with a few short lateral branches, not brush-like in appearance, abdominal palmate seta 1- Abdominal segment I non-palmate or very weakly palmate, Seta 1, 2- Prothorax tubercles separate; seta 3- Metathorax non-palmate or very weakly palmate, Seta 2, 3- Cephalon simple, finely or distinctly frayed, anal papillae normal, abdominal palmate filaments ≤ 0.5 length of blade, Seta 3, 4- Cephalon simple or occasionally bifid, never branched, Seta 2, 3- Cephalon smooth or finely frayed, abdominal seta 1- Abdominal segment II strongly palmate, Seta 3- Cephalon finely frayed, seta 4- Cephalon length > 3- Cephalon, basal tubercle of seta 1- Prothorax weak (Fig. 2) (104).

### The *Anopheles superpictus* s.l. species

*Anopheles superpictus* s.l. acts as a main malaria vector (e.g. in Lorestan) or secondary vector (e.g. Sistan and Baluchistan). A survey has been conducted in order to study on different morphological and genetic variation of *An. superpictus* populations in Iran. Mosquitoes of *An. superpictus* were collected from the Ardebil, Lorestan, and Sistan and Baluchistan provinces in July–September in 2004 using different collection methods. After species identification and morphological study on larvae and adults, the sequence variation of ITS2-rDNA and mtDNA COI-COII fragments were analyzed using PCR-RFLP and PCR-direct sequencing assays. Results showed that there were significant differences in morphological and genetic characters within and between populations. Digestion of COI-COII fragment using AluI enzyme demonstrated various haplotypes indicating intra or inter-species variation. Totally 4 haplotypes were observed between specimens/populations in 708bp of COI gene. Totally, the rate of variation among populations was 12.3%. This rate was 2–5% for within Sistan and Baluchistan populations. According to phylogenetic analysis of the COI sequences, *An. superpictus* populations in Iran constructed 2 main groups including: 1) southeast populations (Sistan and Baluchistan and Kahnooj) and 2) central and northwest populations, each group putatively representing one species. Analysis of ITS2 fragment also revealed highly diverged populations representing at least three putative separate species designated as X, Y, and Z (X and Y in Baluchistan and Z in other provinces). The rate of genetic variation in ITS2 was 27% in which respectively 0.27%, 0.05% and 26.68% corresponded to 5.8S, 28S, and ITS2 regions. Phylogenetic analysis also revealed two main groups including three branches, each one putatively representing one separate species. In spite of having a high genetic and morphological variation among specimens, there were no relationship between phenotypic and genetic variations, assuming that other parts of genome is responsible for the variations. The rate of genetic variation in COI and ITS2 regions was far more than that has been seen in other species s.l. (105).

### Flight ranges

Flight range of this species was about 2–2.5 kilometers (observations in Tabas, Birjand). At the beginning of hibernation it reached to 4km (99).

### Irritability tests

The irritability levels of *An. superpictus* s.l. against diagnostic dose of dichlorodiphenyltrichloroethane (DDT) insecticide were measured in a conical exposure chamber according to WHO methods in Isfahan in 1963. The results of irritability tests showed that DDT had the high irritancy effect against *An. superpictus* s.l. (106).

### The time of blood feeding

The activity of this species started at about 20pm and reached to peak point at about 21.30pm. The most brutal is in the first half and with low density in the second half of the night and in the morning it was close to zero. The most biting activity in Bazaft, Farsan, Chahar Mahal Bakhtiari in 1999 was in 22–23pm and 3–5am (110–111).

### Seasonal activity

Researches have been conducted from early October to April 1953–1955 to determine *An. superpictus* s.l. seasonal activity. *Anopheles superpictus* s.l. adult population abundances for the entire period of August–September were high, but during the October, it began to decrease. The last collection was conducted in the third week of October. Even though space spray collection using Pyrethrums (PSC), that was applied at homes, very few adult were collected in each station during the months of November–February. Based on information about the larvae collection and dissected adults, it has been concluded that this species didn’t enter to hibernation phase in October. It seems that leaving hibernation happened in February, because the females that have been desiccated at this month had high ovarian growth. During the month of May, no larvae were collected. This result showed that although this species ovoposited in February or March, but the eggs were expelled because of abundant rainfall, rapid flow of springs and streams. Changes were not the same during three winters. Winter 1953–1954 had the coldest winter and 1955–1954 was the modest. The numbers of collected specimens to generalize the reliability results were very low. Although the temperature in February, was different from the temperature in the first three months but the collected females showed the high activity both for the ovarian growth and blood feeding. Even in March that this species began its activity, the temperature was more than the last 4 months. It has been justified that *An. superpictus* s.l. entered to hibernation phase because of a physiological cycle caused by cold weather. In Ilam, *An. superpictus* s.l. is the main malaria vector and comprised almost one hundred percent of the collected *Anopheles* from indoors and outdoors (111).

**S**tudies in Kermanshah and Sabzevar indicated that the activity of this species depended on the temperature and the onset of its activity was from mid-June and ended in mid-October. Maximum activity is in early August to mid- September and with the onset of cold weather that is different in various parts of the plains and mountainous areas; after this time it entered to hibernation. Females entered to hibernation phase in mountainous caves and indoors from mid- September in west with storing fat and from October to next April when the weather was good for mosquito activity (Manoochehri, personal negotiation).

In a study in the counties of Pars Abad and Germi in 1997–1998, the activity of this species has been reported during July–November. The maximum abundances of this species was 10.6 per place in the second half of August and with the least frequency was 0.25 number per location in the first half of December in Dykdash and Qeshlaq Beryan villages. Theis species had one peak of activity in second half of August in (112).

In human dwellings, the frequency of *An. superpictus* s.l. in each place was 1.8. In outdoors, *An. superpictus* s.l. had the highest frequency of 6.2 per place in early of August. In human dwellings, its frequency was 1.8 and in outdoors it was 6.2 per each place with the highest abundance in the early of August. The highest monthly activity of *An. superpictus* s.l. in Dykdash Village, Germi, was in the eighteenth of July, 1996. In Dykdash and Agha Mohammad Biglou villages its activity began in eighteenth June and ended in third of December. In the village of Agha Mohammad Biglou the highest activity was in the second half of August. In Dykdash and Gheshlagh Beryan villages, its activity in indoors has been reported during the months of July–November, with the highest frequency of 10.63 mosquitoes per place in the second half of August and the least abundant numbers per place (0.25) in the first half of December. This species hasn’t been collected from plain of Parsabad (112), but studies of Abai and colleagues in 2002 have been obtained very low abundance in this area. The most abundant of this species in the county of Kahnooj was during September and October (113).

Activity of *An. superpictus* s.l. had one peak point in its curve. This species had incomplete hibernation. Hibernation starting differed in cool weather in different plains and mountainous areas. Based on different studies in the North of Khorasan, Birjand, Kazeroon, it has been concluded that in appropriate thermal conditions and activity of *Anopheles*, this species is capable to transmitting *Plasmodium vivax* after the second gonotrophic cycle. Studies conducted in 1996, in the county of Aligoodarz showed that larvae of this species began its activity in May with the peak activity in September and in October it reached to zero (103, 114).

### Species composition

Fauna and ecology of vectors in the county of Farsan, Bazaft, Chahar Mahal Bakhtiari, in human dwellings, indicated that *An. superpictus* s.l. comprised 44.2% of the collected *Anopheles* populations. In animal habitats it comprised 63.7% of the total collected *Anopheles*. In outdoors, *An. superpictus* s.l. comprised 77% of the populations. It has been collected generally in high population density from outdoors. In Hussain Abad village, of the 243 mosquitoes collected in night biting, 165 of them (67.9%) were *An. superpictus* s.l. In the village of Mazi, a total number of 356 *Anopheles* collected with the method of night biting, *An. superpictus* s.l. comprised 246 (69.1%) of the *Anopheles* population. In the village of Hussain Abad, of the 254 collected *Anopheles* in pit shelter the number of 180 of them (70.8%) were *An. superpictus* s.l. (116). The highest biting activity of *Anopheles superpictus* s.l. in Bazaft, Farsan, Chahar Mahal Bakhtiari in 1999 was occurred in 22–23pm and at 3–5am (115).

*Anopheles superpictus* s.l. was the most important vectors in Siahoo area, Southern Iran. Entomological studies conducted in this district showed that of the total collected larvae and adults’ mosquitoes, the *An. dthali*, *An. stephensi*, *An. superpictus* s.l., *An. fluviatilis* s.l. and other *Anopheles* populations had the most frequencies of 37%, 55%, 1%, 2% and 5%, respectively. This species has been found in the mountainous region and played an essential role in disease transmission (116).

In the studies, in Bandar Abbas it has been identified that the most frequency of adult Anophelini were *An. stephensi*, *An. dthali*, *An. fluviatilis* s.l. and with the low density *An. culicifacies* s.l. and *An. superpictus* s.l., respectively. The last two species were collected just in night biting (117).

### Distribution, anthropophilic index, sporozoite rate and behavior of adult *Anopheles superpictus* s.l.

This species had a major role in mountainous region. It had a role in malaria transmission in Hormozgan Province. Combination of malaria vectors in coastal plain and mountainous area of the province was different. The main vector in the coastal plain was *An. stephensi* and the secondary vector was *An. dthali*. In the mountainous region, beside the mentioned species, *An. fluviatilis* s.l., *An. culicifacies* s.l., and *An. superpictus* s.l. had a role in disease transmission. In mountainous areas, because of the presence of several outdoor borrows and exophilic behavior of some vectors, vector eradication encountered many problems and was effective only in lowering the population vector (107).

Anthropophilic index using ELISA with the blood spot prepared from female *An. superpictus* s.l. showed the Anthropophilic index between 28.5–3.7% and the feeding of cattle and other animals were 19.7%. The night bite on prey animals (cows and calves) in the village of Hussein Abad and Namazi villages indicated that 55.6% and 66.2% of them were *An. superpictus* s.l. Based on this study it appeared that this species had zoophilic behavior. In this study *An. superpictus* s.l. had two peaks of activity, in the early hours of the night and the other in 3–5a.m. and its frequency in first peak was more than the second peak. A similar result has been obtained in previous studies (99, 118).

The numbers of 200, *An. superpictus* s.l. were used to determine parity and age-structure of the *An. superpictus* s.l. populations. Parous percent in the first period of the seasonal activity was observed in 26% and its peak of activity was 44%. This species in the study area naturally preferred outdoors to indoors. As in some parts of Iran, especially in mountainous areas his species was found infected to sporozoites (Sarakhs and Kermanshah) and is responsible for malaria transmission and having anthropophilic index, so it could be a vector (119).

About 16% of the total larvae collected in the months of April till November in 1994 in Lenjanat, Isfahan, belonged to this species. Larval activity in its larval habitats began from mid- June and late July with the frequency of 1 larva per ladle. The frequency was constant until the end of August, but its activity decreased in abundance from this time, so that by the mid-September it has been reduced to zero. This species has been collected in light traps with the frequency of 0.21% and adult activity increased simultaneously with larval activity in mid-July and has reached to its activity with the frequency of 1 mosquito per light trap. Its activity decreased in a way that it reached to zero in late August. This is species was mainly endophilic in the area and preferred blood feeding from animals to human. Total collection of this species in light traps and night biting methods in March to November 1994 in Lanjanat, Isfahan was very low (120).

This species has been collected in indoors of Germi county including villages of Ojagh, Zahra Kandi (Ojaroud), Agha Mohammad Beiglou, Dykdash, Qeshlagh Beryan (Angoute gharbi), Aq Dash (District of Barzand). In mountainous region of the Germi, Ardabil including Qeshlagh Beryan and Dykdash, the frequency of *An. superpictus* was 10.63 mosquitoes per place and had a wide distribution (112).

Two species, *An. superpictus* s.l. and *An. maculipennis* s.l., have been distributed in larval habitat studied in Mashhad and suburbs. *Anopheles superpictus* s.l. with the frequency of 94% were more frequent in comparison to *An. maculipennis* s.l., and were collected from several larval habitats including fresh water, non-polluted and largely stagnant waters with low flows. South of Mashhad, places like ponds, even with non-infected freshwater ponds, Astan Quds Razavi farms and its surroundings, highways and Qasem Abad village were places that two species of *An. superpictus* s.l. and *An. maculipennis* s.l. were collected (121).

*Anopheles superpictus* s.l. in villages and tribal areas in Bazaft, Farsan, Chahar Mahal Bakhtiari had a wide distribution. Frequency of adults and larvae were 67.2% and 78.9%, respectively. In the studied villages, *An. superpictus* s.l. comprised 63% to 100% of collected *Anopheles*. This species was generally the dominant species in the area. The larvae of this species have also been collected from the height of 2500m above sea level (the Sartang Sarveh Moorez) while collecting of this species has been reported as high as 2,000 meters above sea level. It has been reported that this species had exophilic behavior in the mountainous areas of Sarakhs and Kermanshah. In Farsan, this species had more frequency in outdoor shelters such as mountains’ crack, caves or under large rocks, under the bridge and river wall and like the other parts of its distribution it was exophilic. Its seasonal activity began from second half of the July and continued till October with the highest frequency in August in outdoors. Adult highest frequency in the study area was 6.2 *Anopheles* per place and highest frequency of larvae in 10, ladle was 33.7. In the studied area, *An. superpictus* s.l. was found mainly in cracks of the trees, mountains groove, caves and cracks between the rocks that were used for flat -out accommodation of tribal, pit walls, rivers, canals and springs, under bridges, human dwellings, closets and behind furniture, in dark storage, networking within the outdoor, and shower stalls. Larval habitat of this species was mainly in margins and riverbeds, streams flow in grasslands springs, animal footprints, holes and stagnant water around the road. Habitats were more dirt floor in a meadow.

The larval habitats were found more in burrows in shaded and sunny waters in most places. In a pit shelter study in Hussein Abad village in Farsan, Chahar Mahal Bakhtiari, of the 78.5% of the collection belonged to *An. superpictus* s.l. that indicate that this species was exophil and most prevalent species in the study areas. With regard to the eradication of this species because of its exophilic property, insecticide repellency, it could not stop disease transmission just using residual spraying and must use environmental improvements, using larvicides and case finding and integrated pest management (115).

In survey that has been conducted in 1971 in this area, results showed that frequency of adult *An. superpictus* s.l. in Germi County has been increased. This species had a salient activity with *An. sacharovi*. Increased abundance of this species that is of main malaria vectors in Iran, is most serious threat to the residents (122–123).

### Human biting rate

In Hussain Abad village, the bites per person in a night were 1.1. This figure for each person in Nazi village was 1.4 (115).

### Blood feeding preferences

This species was zoophage, and both endo-exophilic. Blood feeding preference of human varied depending on geographic location and has been recorded from 5% (124–125).

In Iran, the human blood index for this species has been determined 22.4% and the sediment test of fed females collected from caves have shown 4.1% the human Blood Index (119, 125).

Blood feeding habits, resting and longevity of *An. superpictus* s.l. in central plateau and south parts of Iran is like *An. fluviatilis* s.l.. The results of the precipitation test in different areas of Iran, for anthropophilic index have been reported between 4.8 to 21.4% (124)

In a research study in Lorestan Province to determine blood meal type, fed by this species using ELISA, of the 1117 tested blood spots, the number of 128 specimens had positive ELISA for human blood and thus the anthropophilic index of this species in this study has been reported 11.4% (126–130).

*Anopheles superpictus* s.l. fed on animal and human and preferred blood feeding in areas with large groups of hosts than sporadic hosts. It preferred large body size hosts to small hosts. This species fed on different hosts, when it got dark or night, even in places both inside and outside areas. The host-seeking behavior of this species for blood feeding was highly active, and could search many buildings in search of food, and had a high mobility during blood feeding.

Results of anthropophily and zoophily tests in Bazaft, Farsan in Chahar Mahal showed that out of the 92 blood samples, 28.4%, 39.1%, 32.6% of the specimens’ belonged to human, cattle and other animals, respectively. Investigations in Khorasan showed that human blood feeding on human varied between 65–76%.

Studies conducted from May till November, between 1997 and 1998 by total catch in three indoor places (human, animal, and barn) and one outdoor place, cave, indicated that this species preferred feeding on domestic ruminants and animal than man. The indexes of blood feeding of fed females by ELISA in all places were 1.02%, and in outdoors (Cave), barns, 1.45% and 2.82%., respectively. The human blood index of *An. superpictus* s.l. has been calculated zero, in human and animal places. The observations in the studied villages showed that most of the people because high temperatures at night sleep in open spaces (courtyard of the houses) and some of them slept in nets. Livestock that their numbers were much higher than the number of people in the house that were kept in the open spaces. Unfed mosquitoes after entering the houses preferred feeding on animals than humans. Then they rest followed proper shelter (human indoors, animal, barn or outside shelters). These reasons may explain proper zero coefficient of blood feeding on human in indoors (118). Anthropophilic index for the *An. superpictus* s.l. in its maximum frequency was very low (9.09%) (131).

### Longevity of the *Anopheles superpictus* s.l. populations

Various studies (regions of Birjand-North Khorasan, Kazeroon, etc.) has shown that in appropriate temperature conditions, *An. superpictus* s.l. after the second gonotrophic cycle is capable of transmitting *Plasmodium vivax*. In nature there is up to 5 gonotrophic cycles. It has been observed that *An. superpictus* s.l. for the first laying period was usually required twice blood feeding. Of the 80, *An. superpictus* s.l. (58.7%) were nulliparous, 33 of them were diagnosed parous (41.3%). Result of the dissection of *An. superpictus* s.l. females, the rate of parous females was found to be 48.5% in 1^st^ gonotrophic cycle, 36.5% in 2^nd^ gonotrophic cycles and 15.1% in 3^rd^ gonotrophic cycles. Basically *An. superpictus* s.l. in this area is most dangerous age in September. Epidemiological studies showed that in Birjand and Kazeroon, *An. superpictus* s.l. was an unstable vector. Desiccated salivary glands of *An. superpictus* s.l. in Aligoodarz, showed no sporozoites (103).

### Sustainability index

Epidemiological studies in Birjand and Kazeroon showed that *An. superpictus* s.l. is an unstable vector (stability index less than 0.5) and the executive observations support this theory. This means that the use of residual insecticides can easily control this species (excluding the exophil population).

### Adult shelters

In Iran, a significant population of this species rests in human and animal dwellings including mud rooms, shed, stack, barns, straw, tents and mud stables, rick, animal sheds, poultry nests. Some of the population of this species, especially in the West (Kermanshah) and the East (Kashafroud region) didn’t enter to human habitations and has been collected from outdoors including cave, inside grain storage, cracks in the mountain rock, earth fissures in the walls of the rivers, the aqueducts, under the bridge and artificial pits (99, 132–133).

*Anopheles superpictus* s.l. larvae were collected from villages including Hoomeye markazi, Roodan, Sandarak, Sirik, Dej Olia River, Dej Sofla River and Bashagard. This species that comprised 1.3% of the total percentage of collected larvae periodically was active during at least 6 months of the year. This *Anopheles* has been collected from temporary and stagnant waters, 62.5% and 100%, respectively. It has been collected with low percentages from permanent waters. This species could ovoposite in water nests that the water depth was low, with or without plant, sunny or semi-shade. The larvae of this species prefered larval habitat with soil or sandy in their bed along the rivers (to 80%), wetland, grasslands and fresh waters. It chose fresh and clear waters for ovoposition (50%). *Anopheles superpictus* s.l. larvae have been collected together *An. stephensi*, *An. fluviatilis* s.l., *An. dtali*, *An. thurkhudi* and *An. multicolor* in the same larval habitat. In some west and south west provinces of Iran, *An. superpictus* s.l. larvae has appeared from temporary and stagnant waters with the frequency of 40% and with low density from permanent waters with or without plants. The larvae of this species in Kurdistan began their activity in mid-June and ends in mid-October. The peak activity is from early August to mid-September, and its activity dependent on temperature.

The larvae of this species have been collected from the cities of Islamabad-E-Gharb, Paveh, Qasr-e Shirin, Harsin (Bakhtaran) in 1984–1985. In Bakhtaran the maximum abundance of larvae were found in July and its activity were reported from June to November. In Ilam the activity of 3^rd^ and 4^th^ larvae began from June to September, with most activity and abundances in July. In Khuzistan Province, activities of 3^rd^ and 4^th^ instars larvae were from May to October and with its peak of activity in July and August. In Germi and Pars Abad, the larval habitats included along the Dare Roud River, rice fields, small cavities and holes in the river beds (113).

In Bazaft, Farsan, Chahar Mahal Bakhtiyari, of the 6 villages and 4 tribal regions, *An. superpictus* s.l. comprised 44.2% of the *Anopheles* populations in human dwellings. In animal places *An. superpictus* s.l. comprised 63.7%, of the *Anopheles* populations and in outdoors it was 77%. Totally, this species has been collected from outdoors. In Hussain Abad village, 243 mosquitoes were collected by night-biting method that 165 of them (67.9%) were *An. superpictus* s.l. In this village in Farsan, of the 254 *Anopheles* mosquitoes collected in pit shelters, 180 (70.8%) of them were *An. superpictus* s.l.. Frequency of *An. superpictus* s.l. were 162 (66%) larvae collected from the tribal wattle, 130 (100%) from Shermak, 168 (66.7%) from tribal regions of Labad and in tribal regions of Tashnavi it was 181 (63%) (116).

Studies conducted from May till November, between 1997 and 1998 by total catch method in three indoors (human, animal, barn) and one outdoor (cave) in the province of Lorestan showed that of 1661 collected Culicidae mosquitoes, the 1632 (98.25%) were *Anopheles* genus, and among them the total number of 1630 (99.3%) of the collected *Anopheles* were *An. superpictus* s.l. 631 (50.8%) collected female *An. superpictus* s.l. were freshly feeders (119).

### Malaria transmission potential

In some parts of Iran, especially Kashaf roud in Khorasan and Kermanshah it was discovered that despite eradication programs, malaria transmission by this species has not stopped (Mesghali unpublished observation, 1968). Vectorial capacity is a measure of epidemiology in different geographical regions that is used to determine the malaria transmission risk. Vectorial capacity is affected by three factors including anthropophily, longevity, and mosquito vector population density. In Iran, sporozoite rate has been reported 0.9%. Desiccation of salivary glands of *An. superpictus* s.l. in Khorasan, Kermanshah, Kazeroon and Masjed-E-Soleiman showed that almost 1.2% of the desiccated specimens were infected to sporozoite. In Tabas 4.6% of the specimens and in Kazeroon 0.65% of the specimens were infected with sporozoite and 0.7% of them were infected to oocyte. These studies indicated that *An. superpictus* s.l. was of the major malaria vectors in the central plateau and the southern slopes of the Zagros Mountains of Iran (128).

*Anopheles superpictus* s.l. has been identified as a main malaria vector in all regions of central Iranian plateau. In addition, this species had significant role in maintaining malaria as a second or third vector in mountainous and foothills of southern Zagros range. In 255 desiccated specimens in Birjand in 1961, the sporozoite index was 4.7% (accidental sampling). Desiccation of 411 *An. superpictus* in the mountainous region of Shapoor (Kazeroon) in 1960, infective index was 0.65%. In Bazaft, Farsan, dissection of 200 *An. superpictus* to determine the number of parous and nulliparous showed that the parous rate were 26%, 46% and 40% in late July, August and September, respectively. A total of 126 *Anopheles* salivary glands and stomach were dissected and none of them was found infective to sporozoite and oocyst (116).

### Larval habitats

This species can be found in beds of rivers that are drying in hot climate of the summer. This species have been collected from sandy margins of the river (Gamasab and Chamkhal rivers in Kermanshah Province).

Captures of 2^nd^ (early instars), 3^rd^–4^th^ (late instars) instars and pupae have been done for *An. superpictus* s.l. immature abundances in different agro-ecosystems in Iran. *Anopheles superpictus* s.l. larvae are associated with gravel or fresh waters of swamplands and marshes, and pebble river and stream beds in shallow, slow-flowing clear water in full sunlight, small rivers or streamlet, holes, pits and natural dams, small pools within or next to river beds and streams, storage tanks, broken pitchers, empty cans, water reservoirs, rice fields and associated irrigation canals, natural grassland, large rivers that makes pools, animal hoof traces, tires, small pools within or next to river beds, are suitable as aquatic habitats.

Preferred water temperature for ovopositing of this species in day time, usually reached to 35 °C to 38 °C. In 30 °C, the larval period lasted 11 days. However, at temperatures between 19 °C to 20 °C, the larval period has been estimated at 24 days. Frequency of 3^rd^ and 4^th^ instars of *An. superpictus* s.l. in each ladle from April till November 1994 in Lanjanat, Isfahan in late July, mid-August and late August were 0.1, 0.1 and 0.1, respectively and in the remaining months was zero (122).

Ecological characteristics of larval habitats of *An. superpictus* s.l. in 24 larval habitats in Lorestan Province showed richness of species composition that is indication of the importance of land use patterns on diversity of larval habitat types that included *An. superpictus* s.l., *An. turkhudi*, *An. stephensi*, *An. dthali*, *An. sacharovi*, *An. claviger* and *An. martri. Anopheles superpictus* s.l. larvae were collected from standing or temporary and especially from still waters. This species was active in the waters with aquatic vegetation on their surface or outside of them. More than 45% of the specimens were collected from habitats without any aquatic vegetation. In 87.5% cases this species oviposit in the sandy and soil beds habitats with fully sunlight. This species prefers clear and fresh waters. Their larval habitats were close to rivers (53.2%), lakes, grasslands, holes, streams, and river beds (46.8%). In man-made larval habitats, 87.5% larval habitats were found in rice fields and 12.5% were in cement grooves. Larval peaks of activity have been reported from mid-July till second half of the October. 67.8% of the larval habitats were standing and 32.2% of them were temporary. 62.6% of the larval habitats were still waters and rests of them were running waters. In this survey from the 115 larval habitats, 88.7% of the specimens were collected from sunny habitats. This species preferred to oviposit in sandy bed larval habitats (55.7%), mud (40.8%) and cement canals (3.5%).

These species were mainly found in habitats without vegetation (46.9%), vegetation outside of the water (36.5%) and the rests of them in vegetation on the surface or under the water. In Lorestan Province, larval habitats were found in agro-villages s.l.es of Pishkouh Zalghi (villages of Baghe lotfian, Kizan Darreh, Kakolestan), Farsash (villages of Farsash, Homa and Ivaj), Barbaroude Shargi (villages of Chaman Soltan (Shahrake Emam), Ab Barik, Dehnou Khaje), Pache Lake Shargi (villages of Dehe nasir, Sour, Choghatar), Mahrou (villages of Margsar and Chal Ghale).

In Bazaft, Farsan, Chahar Mahal Bakhtiari in 1999, in the tribal region, activity of different species of *Anopheles* in ten studied villages were investigated 120 times. A total number of 2543 larvae were collected from different larval habitats including river margins, stagnant water, grasslands and springs. In tribal regions a total number of 162 larvae were collected that 66.6% of them were *An. superpictus* s.l.. In Labeh of the 168 collected larvae, the frequency of *An. superpictus* s.l. was 66.7%. In Shermak area of the 130 collected larvae 100% of them were *An. superpictus* s.l.. In Teshnavy that is a tribal area of the 181 collected larvae in 12 times, 63% of them were *An. superpictus* s.l.. *Anopheles superpictus* s.l. larvae with the abundance of 33.7% in 10 ladles had the highest density.

### Adult susceptibility tests

**Fig. 3.**
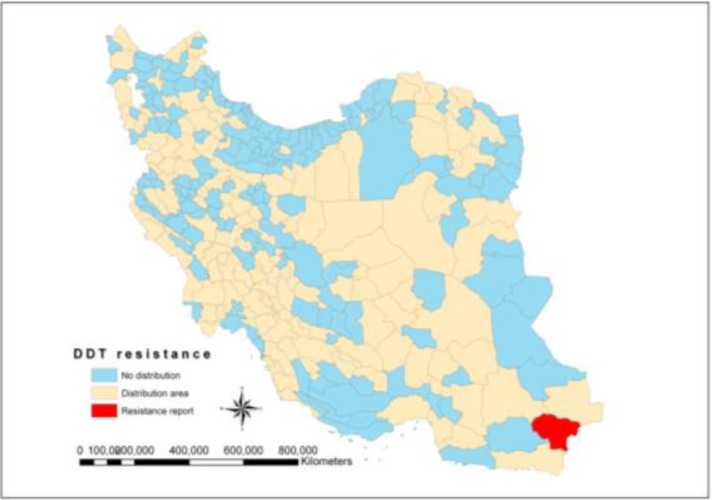
Mapping status of *Anopheles superpictus* to DDT in Iran

The insecticide susceptibility tests on field adults and larvae were performed in 1972–1975 on the *An. superpictus* s.l. collected from different parts of the country including mountainous, temperate and desert areas including Isfahan, Kermanshah, Khorasan, Kurdistan, and Ilam provinces. For the adult tests, female *An. superpictus* s.l. were exposed to discriminating doses of OC, OP and PY insecticides including D.D.T. (mortality rate: 99.4%), malathion (mortality rate: 100%), lambdacyhalothrin (mortality rate: 100%) and dieldrin for 60min. The effects of these pesticides were evaluated in accordance with WHO standards, using mortality rates after 1-hour exposure followed by monitoring over a 24-hour recovery period. The adult susceptibility tests using WHO criteria (98–100% mortality indicating susceptibility and < 98% mortality indicating resistance), showed that *An. superpictus* s.l. was susceptible to all the tested insecticides (107–109). In Ilam Province, in 2002, the percentage of mosquitoes knocked down at 60 minutes were 100%, 99% and 98% for lambdacyhalothrin, malathion and DDT, respectively (111). Table 1 and Figure 3 show the resistant of species to DDT and susceptible to other insecticides in the country.

**Table 1.** Status of insecticide resistance in *Anopheles superpictus* from Iran, 1957–2016

**Insecticide**	***Anopheles superpictus***
**DDT 4%**	R
**Dieldrin 0.4–4%**	S
**Malathion 5%**	S
**Lambdacyhalothrin 0.025–0.1%**	S

S= Susceptible

R= Resistant

## Discussion

Malaria remains an important parasitic disease in different parts of Iran, despite decades of organized malaria control activities. Malaria is particularly prevalent in tropical provinces including Sistan and Baluchistan. Iran remains highly conducive to vector borne diseases due to geography, and uncontrolled population movement. The *Anopheles* (*Cellia*) *superpictus* s.l. is important malaria vectors that have been found in different provinces of Iran. *Anopheles superpictus* s.l. represent important vectors of malaria throughout the Palearctic Region, including Iran. This s.l. complex contains at least three closely-related sibling species that can be differentiated based on distinctive genetic characters. Current evidence shows that climate variability has a direct effect on the epidemiology of vector-borne diseases including malaria). The most influence of climate change on disease transmission is likely to be monitored at the extremes of the range of temperatures at which transmission develops. Malaria is of the most dangerous vector-borne diseases in the tropical and subtropical areas. Health risks because of climatic variability will be different among countries that have developed health bases and those that do not (134). Patterns of human settlement in the various areas will affect disease tendency s.l. complex. It has evidenced important anthropogenic ecosystem changes and it contains a great frequency and diversity of mosquito-breeding sites and as a result hosts large mosquito populations, and in areas of endemic malaria. Results indicate that, environmental modifications and changes in the economic, social, and cultural environments can have tough and rapid influences on mosquito populations, climate variability, and drug resistance. These factors influence on biting, survival, and reproductive rates of vectors (135–143). We recommend new research on different aspects of *An. superpictus* in the future for decision making.

## Conclusion

Understanding of all bioecology of malaria vector will guide the scientist and decision makers for appropriate diseases control.
